# Uptake Rate of Cationic Mitochondrial Inhibitor MKT-077 Determines Cellular Oxygen Consumption Change in Carcinoma Cells

**DOI:** 10.1371/journal.pone.0037471

**Published:** 2012-05-17

**Authors:** John L. Chunta, Kerry S. Vistisen, Zeinab Yazdi, Rod D. Braun

**Affiliations:** 1 Department of Anatomy and Cell Biology, Wayne State University School of Medicine, Detroit, Michigan, United States of America; 2 Barbara Ann Karmanos Cancer Institute, Wayne State University, Detroit, Michigan, United States of America; University of South Alabama, United States of America

## Abstract

**Objective:**

Since tumor radiation response is oxygen-dependent, radiosensitivity can be enhanced by increasing tumor oxygenation. Theoretically, inhibiting cellular oxygen consumption is the most efficient way to increase oxygen levels. The cationic, rhodacyanine dye-analog MKT-077 inhibits mitochondrial respiration and could be an effective metabolic inhibitor. However, the relationship between cellular MKT-077 uptake and metabolic inhibition is unknown. We hypothesized that rat and human mammary carcinoma cells would take up MKT-077, causing a decrease in oxygen metabolism related to drug uptake.

**Methods:**

R3230Ac rat breast adenocarcinoma cells were exposed to MKT-077. Cellular MKT-077 concentration was quantified using spectroscopy, and oxygen consumption was measured using polarographic electrodes. MKT-077 uptake kinetics were modeled by accounting for uptake due to both the concentration and potential gradients across the plasma and mitochondrial membranes. These kinetic parameters were used to model the relationship between MKT-077 uptake and metabolic inhibition. MKT-077-induced changes in oxygen consumption were also characterized in MDA-MB231 human breast carcinoma cells.

**Results:**

Cells took up MKT-077 with a time constant of ∼1 hr, and modeling showed that over 90% of intracellular MKT-077 was bound or sequestered, likely by the mitochondria. The uptake resulted in a rapid decrease in oxygen consumption, with a time constant of ∼30 minutes. Surprisingly the change in oxygen consumption was proportional to uptake rate, not cellular concentration. MKT-077 proved a potent metabolic inhibitor, with dose-dependent decreases of 45–73% (p = 0.003).

**Conclusions:**

MKT-077 caused an uptake rate-dependent decrease in cellular metabolism, suggesting potential efficacy for increasing tumor oxygen levels and radiosensitivity *in vivo*.

## Introduction

Solid tumors are significantly hypoxic [1] because of an imbalance in oxygen supply and demand. Hypoxia negatively impacts the efficacy of various tumor treatments, especially radiation therapy [2], [3]. This well-known effect is directly related to the mechanism of radiation-induced cell death. DNA damage from ionizing radiation is made permanent when molecular oxygen, present at the time of radiation, binds to DNA strand breaks [4], ultimately causing cellular death. Diminished oxygen levels yield cells that are more likely to repair the radiation-induced defects using their DNA repair machinery, escaping this death pathway. Where hypoxia exists, larger radiation doses are required to obtain similar outcomes as achieved under normoxic conditions [1], [4], [5], [6]. Therefore, there has been considerable effort to increase tumor oxygen levels as a means of increasing radiosensitivity [7].

Tumor hypoxia can be reduced by either increasing oxygen supply to the tumor or decreasing the tumor's demand for oxygen. Theoretical modeling has demonstrated that reducing oxygen consumption (demand) is the most effective method [8], [9], [10]. Small decreases in consumption (≤30%) translate into large increases in local oxygen levels. This effect has been demonstrated in conjunction with radiation in a study investigating glucocorticoids as metabolic inhibitors in intramuscular murine tumors [11].

In this study, the delocalized lipophilic cation MKT-077 was investigated as an inhibitor of cellular oxygen consumption, and the kinetic relationship between drug uptake and metabolic inhibition was characterized. This compound had been tested preclinically as a chemotherapeutic agent, where it preferentially accumulated in carcinomas and was extremely cytotoxic in *in vitro* and *in vivo* models [12], [13], [14], [15], [16], [17], [18]. Since the drug accumulated in the mitochondria, its cytotoxicity was originally primarily attributed to mitochondrial damage and nonspecific inhibition of the enzymes of the electron transport chain [15], [16], [18]. This inhibition of electron transport resulted in a decrease in mitochondrial oxygen consumption. MKT-077 was advanced to Phase I clinical trials, but its progression was halted, due to limited antitumor effects and mild nephrotoxicity [19], [20]. Since MKT-077 has been shown to inhibit respiration in isolated mitochondria [16] and has been advanced to clinical trials, it is possible that this drug could be a useful radiosensitizer by raising local tumor oxygen levels.

Although MKT-077 accumulates in mitochondria and inhibits isolated mitochondrial oxygen consumption [15], [16], the relationship between cellular drug uptake and the extent of cellular metabolic inhibition is unknown. Since the plasma membrane and other cellular components can influence drug uptake, it is important to determine MKT-077 uptake and uptake kinetics in whole cells. Our hypothesis was that MKT-077 would be taken up by breast adenocarcinoma cells in a dose- and time-dependent manner and that the inhibition of cellular oxygen consumption would be related to the drug uptake. The results of these studies will guide future *in vivo* characterization of MKT-077 as a possible tumor metabolic inhibitor. Since inhibition of oxygen consumption will increase tumor oxygen levels, MKT-077 could have the potential to increase hypoxic tumor radiosensitivity.

## Methods

### MKT-077

MKT-077 (1-Ethyl-2-{[3-ethyl-5-(3-methyl-benzothiazolin-2-yliden)]-4-oxothiazoli-din-2-ylidenemethyl}pyridium chloride) was a generous gift of Dr. Keizo Koya (Synta Pharmaceuticals Corp., Lexington, MA). MKT-077 was dissolved in saline (1 mg/ml), and MKT-077 calibration solutions were prepared by serial dilution. Solution absorbance was measured at 495 nm [15], using a BioRad SmartSpec 3000 spectrophotometer (Hercules, CA). The resultant standard curve was used to determine the MKT-077 concentrations in subsequent experiments.

### Growth of R3230Ac and MDA-MB231 Cells

The R3230Ac rat breast adenocarcinoma cells used in this study were a gift from Dr. Mark Dewhirst (Duke University Medical School, Durham, NC). The R3230Ac cell line spontaneously arose from a rapidly growing lactating rat mammary tumor (R3230AB) in the Fischer 344 rat [21]. Cells were grown under standard incubator conditions in Dulbecco's Modified Eagle Medium (D-MEM), with high glucose, L-glutamine, and sodium pyruvate, supplemented with penicillin-streptomycin and 10% fetal bovine serum. After growth to ∼80–90% confluency, cells were harvested and resuspended to 1×10^6^ cells/ml in phenol red-free D-MEM with identical supplementation. Since MKT-077 is orange, phenol red-free media was used to limit background spectroscopic interference.

A subset of experiments was also performed on MDA-MB231 human breast carcinoma cells, which were purchased from the American Type Culture Collection (ATCC, Manassas, VA). The MDA-MB231 breast cancer cell line was generated from a pleural effusion in a 51 year-old patient at M. D. Anderson Cancer Center in 1973 [22]. Cells were grown and maintained in Leibovitz's L-15 media (LL-15) plus antibiotics and 10% fetal bovine serum.

### *In Vitro* MKT-077 Uptake

A 15 ml suspension of cells was placed in a water-jacketed chamber equilibrated to 37°C and was gently mixed with a small magnetic stir bar. After obtaining a negative control sample, MKT-077 was added to the system to bring the concentration to 2, 4, or 6 µg/ml. 1.5 ml samples were taken immediately and every 30 minutes thereafter over two hours. Samples were centrifuged, washed with PBS, and lysed with 200 µl ethanol. Samples were then re-centrifuged, and the lysate was analyzed spectrophotometrically at 495 nm. In addition to the 1.5 ml sample, a 100 µl aliquot was taken from the chamber to determine cell viability using the trypan blue exclusion technique [23]. The chamber and samples were continuously protected from light, since MKT-077 is photosensitive [24].

### Model of Cellular MKT-077 Uptake

As detailed in the Appendix S1, cellular uptake of MKT-077 was modeled by assuming that drug uptake is driven by a combination of the concentration gradient and the electrical potential gradient across the plasma membrane. By using the constant field assumption of Goldman [25] to describe the transmembrane drug flux, the intracellular drug concentration can be described at any time t by:
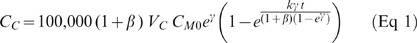
where C_C_ = total intracellular drug concentration (ng MKT-077/10^5^ cells), β = equilibrium constant (dimensionless) relating the mitochondrial MKT-077 concentration to the cytoplasmic MKT-077 concentration, V_C_ = cellular volume (ml/cell), C_M0_ = initial MKT-077 concentration in the medium (ng MKT-077/ml), k = mass transfer coefficient across cell membrane (sec^−1^), and γ = dimensionless parameter related to the plasma membrane potential, ΔΨ_pmem_ (see Appendix S1).

As t approaches infinity, C_C_ reaches a final steady-state value, C_C,∞_ (ng MKT-077/10^5^ cells), and Equation 1 reduces to:
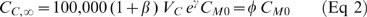
where
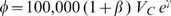
Substituting Equation 2 into Equation 1 yields:
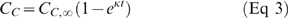
where
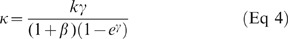
The initial uptake rate [ng MKT-077/(10^5^ cells min)] is given by:
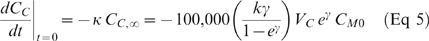



To determine the value of V_C_, images of MKT-077-treated (4 µg/ml) and untreated cells were taken at all time points on an inverted phase-contrast microscope fitted with a digital camera (Q-Color 3, Olympus America, Center Valley, PA) using QCapture Pro software (QImaging Corp., Surrey, BC, Canada). Cell diameters (d) were measured from the digital images using ImageJ software (NIH, Bethesda, MD). Average cell volume was determined (volume = πd^3^/6) using a minimum of 150 cells per time point.

The value of ΔΨ_pmem_ was determined using a modification of the method of Emri et al. [26]. Briefly, R3230Ac cells were harvested and either left in D-MEM medium or fixed in cold 10% buffered formalin for 30 minutes. Then 4×10^5^ cells/ml suspensions of fixed and unfixed cells were exposed to different concentrations of the anionic dye DiBAC4(3) for 30 minutes at 37°C. Aliquots of 4×10^4^ cells at each drug exposure were placed in a 96-well plate. Fluorescent intensity readings were measured spectrophotometrically with excitation and emission wavelengths of 493 and 519 nm, respectively (Spectra Max Gemini EM, Molecular Devices, Sunnyvale, CA). The intensities of the unfixed cells yielded the intracellular concentrations as a function of DiBAC4(3) concentration in the media. Using the intensity curve from the unfixed cells as a calibration, the intensity of the fixed cells was used to determine the extracellular concentration on a cellular basis. The membrane potential was calculated by substituting the ratio of the two concentrations into the Nernst equation. The value of ΔΨ_pmem_ was then used to calculate γ for use in the model equations (see Appendix S1).

Equation 1 was fitted to the uptake data by optimizing the parameters β and k using nonlinear least-squares regression (GraphPad Prism, GraphPad Software, Inc., La Jolla, CA). The goodness of fit was determined by evaluation of the coefficient of determination (r^2^) and the root-mean-square (RMS) error. The validity and uniqueness of β and k were assessed by evaluating the 95% confidence intervals, which indicate how tightly the model has determined these values.

### Estimation of Mitochondrial Membrane Potential

If it is assumed that the vast majority of “bound” MKT-077 is found within the mitochondria, then the relationship between the mitochondrial transmembrane potential, ΔΨ_mit_, and the cytoplasmic and mitochondrial concentrations is given by the Nernst equation and can be calculated as follows at 37°C:
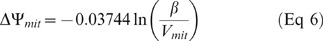
where V_mit_ = relative mitochondrial volume (ml mitochondria/ml cell). V_mit_ was assumed to be 0.10, which is the mitochondrial volume fraction of HeLa cervical cancer cells [27].

### *In Vitro* Measurement of Oxygen Tension in a Closed System

Oxygen tension (pO_2_) was measured using a Clark-type polarographic oxygen electrode (inO2, Innovative Instruments, Inc., Tampa, FL). Saline was equilibrated to 37°C in a water-jacketed chamber, and a calibration line was obtained by measuring the electrode current in saline bubbled with gases of known oxygen concentrations (21%, 5%, and 0% O_2_). The calibration line was used to convert the measured current into pO_2_. After calibration, 5.25 ml of R3230Ac or MDA-MB231 cell suspension (5.25×10^6^ cells) was placed into the chamber. The electrode was inserted into the suspension, which was gently mixed with a small magnetic stir bar, and the chamber was sealed. The oxygen current was recorded at 20 Hz (DaqPAD and LabVIEW, National Instruments, Austin, TX). After a baseline recording (20 minutes), the appropriate volume of MKT-077 stock solution was injected into the system to achieve a concentration of 2, 4, or 6 µg/ml (30 µl maximum). Saline was used for controls. The pO_2_ was then recorded for two hours or until a threshold of 20 mm Hg was reached. This threshold value was chosen, since the consumptive behavior of cells changed below 20 mm Hg, regardless of treatment condition. At the end of the experiment, the cellular MKT-077 concentration was determined spectrophotometrically. Oxygen currents were processed by taking a two-second average every 30 seconds (DIAdem, National Instruments, Austin, TX). Data was analyzed after the currents were converted to pO_2_ using the electrode calibration. Exposure of electrodes to concentrations of MKT-077 up to 6 µg/ml had no effect on the electrode current (data not shown).

### Modeling the Relationship between MKT-077 Uptake and Oxygen Consumption Change

To quantify the effect of MKT-077 on oxygen consumption, we tested numerous models relating the change in oxygen consumption to MKT-077 uptake. It is reasonable to assume that the change in consumption would be related to the amount of MKT-077 sequestered in the mitochondria or the rate at which MKT-077 was taken up by the mitochondria. Since the amount of sequestered drug is proportional to the total cellular MKT-077 concentration, C_C_, the change in consumption can also be related to C_C_, which is a measured variable (see Appendix S1). To determine the nature of the relationship between consumption change and cellular uptake of MKT-077, we tested the following three relationships:
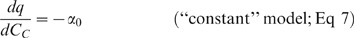


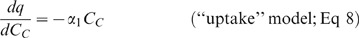


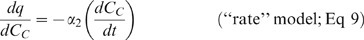
where dq/dC_C_ = the change in cellular oxygen consumption for a given change in cellular MKT-077 concentration {ml O_2_/[(ng MKT-077) min]}, α_0_ = proportionality constant {ml O_2_/[(ng MKT-077) min]}, α_1_ = proportionality constant {[ml O_2_ (10^5^ cells)]/[(ng MKT-077)^2^ min]}, and α_2_ = proportionality constant {[ml O_2_ (10^5^ cells)]/[(ng MKT-077)^2^]}. The models represented by Equations 7, 8, and 9 are designated the “constant”, “uptake”, and “rate” models, respectively.

As described in the Appendix S1, Equations 7–9 can be integrated and simplified to yield the oxygen consumption as a function of time after MKT-077 addition at time t = 0:
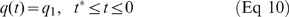


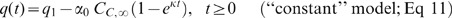


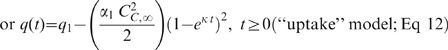


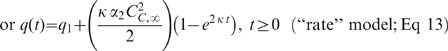
where q_1_ = basal O_2_ consumption rate [ml O_2_/(10^5^ cells min)] and t^*^ = time at which the recording was started.

As detailed in the Appendix S1, these relationships were used to obtain equations describing pO_2_ as a function of time and three unknown parameters (P^*^, q_1_, and α_0_, α_1_, or α_2_): 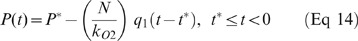


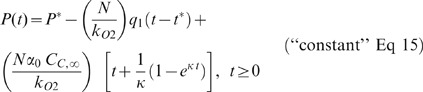


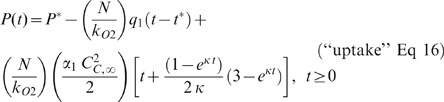


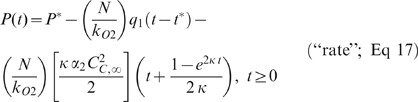
where P = pO_2_ (mm Hg), P^*^ = initial pO_2_ at time t^*^ (mm Hg), N = cellular concentration (1×10^6^ cells/ml suspension), and k_O2_ = oxygen solubility (Henry's Law constant) of the suspension (ml O_2_/ml suspension/mm Hg).

In all cases, the value of k_O2_ was set to 3.0×10^−5^ ml O_2_/(ml suspension mm Hg), which is the oxygen solubility in a dilute protein solution at 37°C [28]. The values of κ and C_C,∞_ were determined from the fits of the uptake data. Using these constants, the experimental pO_2_ data were fitted to Equations 14–17 by optimizing values of P*, q_1_, and α (α_0_, α_1_, or α_2_) using nonlinear least-squares regression (GraphPad Prism, GraphPad Software, Inc., La Jolla, CA).

The goodness of fit was determined by evaluation of the r^2^ and RMS error values. The validity and uniqueness of the parameter values were assessed by evaluating the 95% confidence intervals. All of the data were fitted to these models, except for the saline control data, which were fitted to a straight line, since the rate of oxygen consumption remained constant.

### Statistics

All mean values are reported as means ± SD. Since all parameters for the R3230Ac cells passed normality tests, parametric statistical tests were used for all comparisons. The uptake curves of the three groups were compared using two-way analysis of variance (two-way ANOVA). Model parameter values were compared among the dose groups using one-way ANOVA. Viability data as a function of time within each dose group were compared using one-way ANOVA. Significant differences (p<0.05) were further analyzed using Bonferroni's multiple comparison test to compare values between any two groups. Correlations among parameter values and MKT-077 concentration were determined using linear regression analysis. Regressions were considered significant if p<0.05.

Since the group sizes were smaller for the MDA-MB231 cells, model parameter values were compared among the dose groups using the nonparametric Kruskal–Wallis one-way ANOVA by ranks test.

Since all of the oxygen consumption models contained three unknown parameters, comparisons of the model fits were made using a repeated measures one-way ANOVA test of the RMS errors and r^2^ values. Significant differences (p<0.05) were further analyzed using Bonferroni's multiple comparison test to compare values between any two groups. Differences were considered significant if p<0.05.

## Results

### Effect of MKT-077 Exposure on Cell Viability

The average viability remained well above 90% for all experiments. For the 2 and 4 µg/ml treatment groups, there was no significant effect of MKT-077 exposure time on cell viability (one-way ANOVA, p = 0.064 and p = 0.234, respectively). Thus, there was no significant change in the fraction of viable cells at the end of the observation period (90–120 minutes) compared to the starting viable fraction: 94.3±4.1% vs. 97.1±2.9% (n = 11) for the 2 µg/ml dose and 97.1±1.9% vs. 98.1±1.8% (n = 10) for the 4 µg/ml dose. For the 6 µg/ml treatment group, there was a small, but significant, decrease in cell viability over time (one-way ANOVA, p = 0.0003). At the start of the experiment, the average viability was 98.4±1.7% (n = 8), while after 120 minutes it was significantly lower at 93.9±3.7% (Bonferroni's test, p<0.01). In this group, there were no significant differences between the viability at 30 and 60 minutes compared to the starting value (p>0.05).

### Model of *In Vitro* MKT-077 Uptake

Since cell volume did not change as a function of MKT-077 exposure, V_C_ was set to the average cell volume of 1747±519 µm^3^/cell (mean±SD, n = 718) or 1.747×10^−9^ ml/cell. Plasma membrane potential was measured in five separate experiments, with ΔΨ_pmem_ = −26.1±2.8 mV. Using this value and Equation A4 in the Appendix S1, γ was calculated as 0.9772.

**Figure 1:**
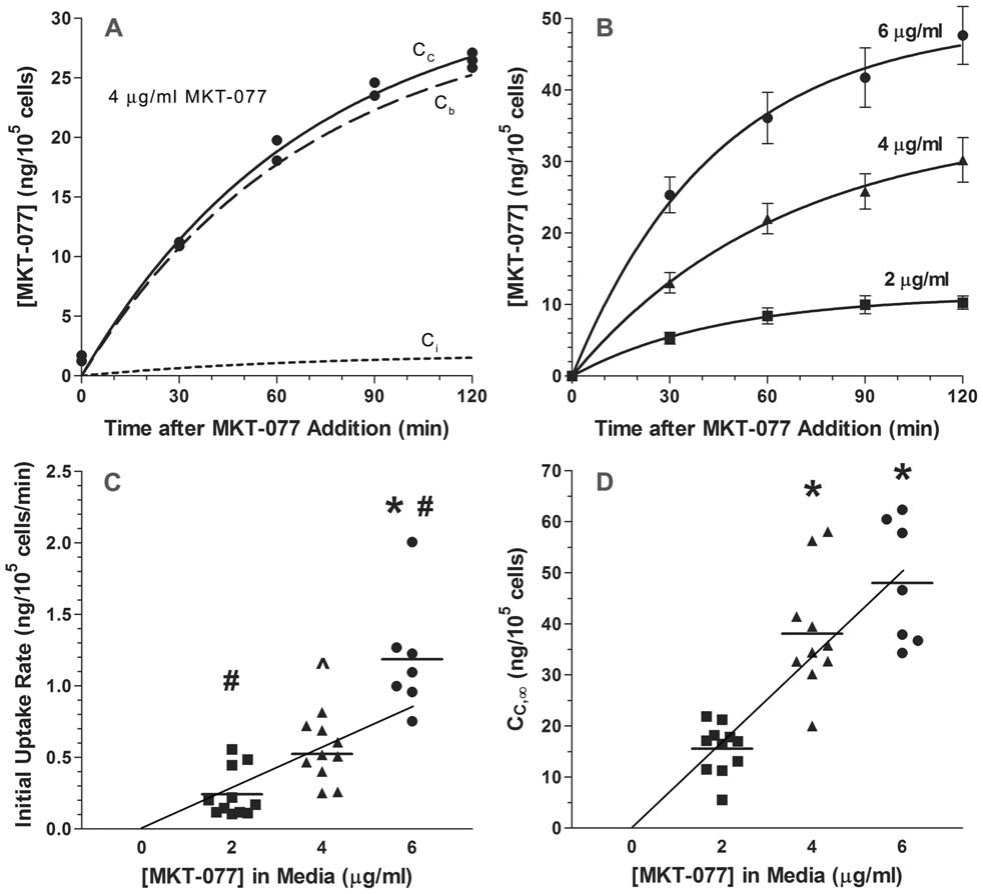
Effect of extracellular MKT-077 concentration on cellular uptake. A) Data from a typical uptake experiment. R3230Ac cells were treated with 4 µg/ml MKT-077 at time 0. Solid line represents total cellular MKT-077 uptake, dashed line represents intracellularly bound or organelle-sequestered drug, and dotted line indicates free drug in the cytoplasm as determined by the model. B) MKT-077 uptake by R3230Ac cells as a function of drug concentration and time. Values are the mean ± SEM. p<0.0001 using a two-way ANOVA. Curves are fits of the mean values to Equation 1. 2 µg/ml: β = 11.1, k = 0.458 min^−1^ (n = 11); 4 µg/ml: β = 18.0, k = 0.502 min^−1^ (n = 10); 6 µg/ml: β = 18.0, k = 0.632 min^−1^ (n = 7). C) Initial drug uptake rate as a function of treatment concentration. Line is calculated from Equation 5 with a slope of 1.44×10^−4^ ml/(10^5^ cells min). D) Steady-state MKT-077 uptake as a function of treatment concentration. Line is calculated from Equation 2 with a slope of 0.00845 ml/10^5^ cells. In panels C and D, the points are staggered along the abscissa for clarity, and the horizontal bars represent the mean values. One-way ANOVA with Bonferroni's Multiple Comparison Tests: * p<0.001 vs. 2 µg/ml value, ^#^ p<0.001 vs. 4 µg/ml value, ^^^ p<0.05 vs. 2 µg/ml value.

Figure 1A shows an example of the fit of the model to experimental data obtained when R3230Ac cells were treated with 4 µg/ml MKT-077. In this example, β was 16.58 and k was 0.427 min^−1^. The 95% confidence intervals were 11.65–21.51 for β and 0.305–0.549 min^−1^ for k. The RMS error was 0.929 ng/10^5^ cells, and r^2^ was 0.994. Qualitatively, there was a relatively rapid rise in the cellular concentration of MKT-077 over the first 30 minutes after exposure. Most of this increase is attributable to the accumulation of drug in the mitochondria or binding to other receptors (long dashed line). Unbound drug in the cytoplasm (dotted line) is extremely low.

Table 1 shows that this model fitted all of the data well. Values of r^2^ ranged from 0.896–1.000, indicating that the model accounted for most of the variability in the data. The average RMS error was less than 1.3 ng/10^5^ cells. The 95% confidence intervals also affirmed the goodness of the model fit.

**Table 1:** Fitted and calculated parameters and goodness-of-fit values for the model fits of MKT-077 uptake.

[MKT-077] (µg/ml)	n	β	k (min^−1^)	r^2^	RMS error(ng/10^5^ cells)	Initial uptake rate (ng/10^5^ cells min)	Steady-state [MKT-077] (ng/10^5^ cells)	ΔΨ_mit_ (mV)
2	11	15.8±5.2 *(5.9–25.6)*	0.446±0.307*(0.161–0.730)*	0.979±0.031	0.621±0.467	0.244±0.168^b^	15.56±4.81	−133.3±11.5
4	10	19.5±6.2(*11.1–27.9*)	0.479±0.172(*0.304–0.654*)	0.991±0.008	1.261±0.817	0.524±0.189^c^	38.09±11.59^a^	−139.6±8.7
6	7	16.2±4.3(*14.2–18.3*)	0.723±0.244 (*0.564–0.882*)	0.996±0.005	1.126±0.620	1.188±0.401^a^^,^ b	48.03±12.10^a^	−135.1±7.3
ANOVA p-value		0.262	0.073	0.197	0.080	<0.0001	<0.0001	0.329
All doses	28	17.2±5.5	0.527±0.268	0.987±0.021	0.976±0.690	----	----	−136.0±9.7

*Values are means* ± *SD. Values in italics are the means of the 95% confidence intervals. One-way ANOVA with Bonferroni's Multiple Comparison Tests:*

a *p<0.001 vs. 2 µg/ml value,*

b *p<0.001 vs. 4 µg/ml value,*

c *p<0.05 vs. 2 µg/ml value.*

### Effect of Extracellular Drug Concentration on MKT-077 Uptake

The mean uptake values for each time point are shown in Figure 1B, along with the fit of the model to these values. R3230Ac cells took up MKT-077 in a dose- and time-dependent manner (p<0.0001, 2-way ANOVA). Treatment of cells with 2 µg/ml MKT-077 (n = 11) resulted in the lowest uptake at the end of the two-hour incubation period (10.4±1.9 ng/10^5^ cells). Cells treated with 4 (n = 10) and 6 µg/ml MKT-077 (n = 7) exhibited higher 2-hour drug levels of 28.7±11.1 and 49.2±10.9 ng/10^5^ cells, respectively.

Uptake analysis using the kinetic model revealed no significant difference between the β values in cells treated with 2, 4, or 6 µg/ml MKT-077 (p = 0.262, Table 1). These β values translated into ΔΨ_mit_ values of −133.3 to −139.6 mV. Similarly, there was no significant difference in the fitted k values (Table 1), although the value for the 6 µg/ml group tended to be slightly higher.

One-way ANOVA analysis determined that there was a significant difference in the initial uptake rates and the steady state MKT-077 concentrations (p<0.001, Table 1, Figures 1C, D). The initial uptake rates for cells treated with different concentrations of MKT-077 were significantly different from each other (Figure 1C, p<0.05). Equation 5 (line in Figure 1C) predicts the initial uptake rate for the 2 and 4 µg/ml doses well, but tends to underestimate the value for the 6 µg/ml dose. Cells treated with 4 µg/ml and 6 µg/ml of MKT-077 each had a higher steady state uptake compared to cells treated with 2 µg/ml (Figure 1D, p<0.001). The line in Figure 1D is the model prediction, as given by Equation 2. The slope, φ, was calculated using the parameters and has a value of 0.00845 ml/10^5^ cells.

### Comparison of Models of *In Vitro* pO_2_ and Oxygen Consumption

**Figure 2:**
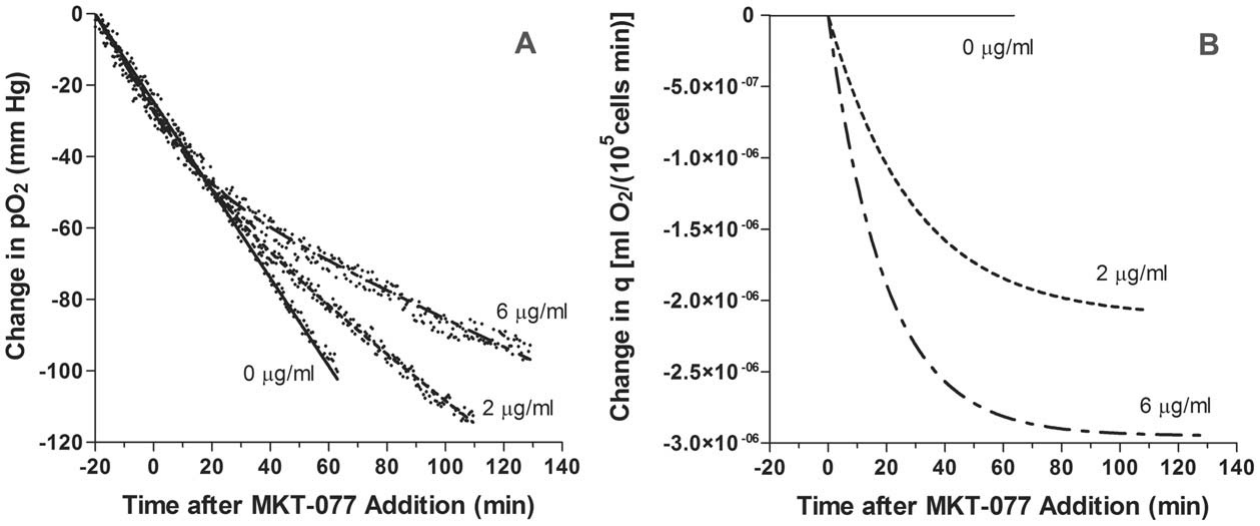
Examples of the inhibition of oxygen consumption by MKT-077 and resultant model fits. A) Change in pO_2_ measured in R3230Ac cell suspension before and after addition of different concentrations of MKT-077 (0, 2, or 6 µg/ml) to air-saturated medium at t = 0 minutes. Points are measured pO_2_ data. Curves are fits of data to the “rate” model (Equations 14 and 17). B) Changes in oxygen consumption, q-q_1_, predicted by the “rate” model (Equations 10 and 13).

To determine the drug's impact on oxygen consumption, 6–7 experiments were performed at each MKT-077 drug level (0, 2, 4, and 6 µg/ml). Examples of individual experiments at the 0, 2, and 6 µg/ml doses are shown in Figure 2A. The points are the recorded pO_2_ values. From −20 to zero minutes (baseline), a linear decrease in pO_2_ was evident in all cases, indicating an unchanging oxygen consumption rate. The slope of the line remained unchanged after addition of saline (0 µg/ml). After 2 or 6 µg/ml MKT-077 was added (t = 0), the curve became nonlinear, indicating a decrease in oxygen consumption (Figure 2A). As the MKT-077 dose increased, the curve became progressively flatter, i.e., oxygen consumption decreased further.

The pO_2_ data from the cells treated with saline were adequately fitted by a straight line (Figure 2A). The pO_2_ data from all 20 MKT-077 dosing experiments were fitted to the three different equations outlined in Materials and Methods (Equations 14–17). Since there was no significant dose effect on the uptake parameters, the overall mean values of β and k (Table 1) were used to calculate κ and Equation 2 was used to calculate C_C,∞_. Therefore, κ was set to −0.017 min^−1^, and C_C,∞_ was set equal to 16.9, 33.8, and 50.7 ng MKT-077/10^5^ cells for the 2, 4, and 6 µg/ml doses, respectively.

**Figure 3:**
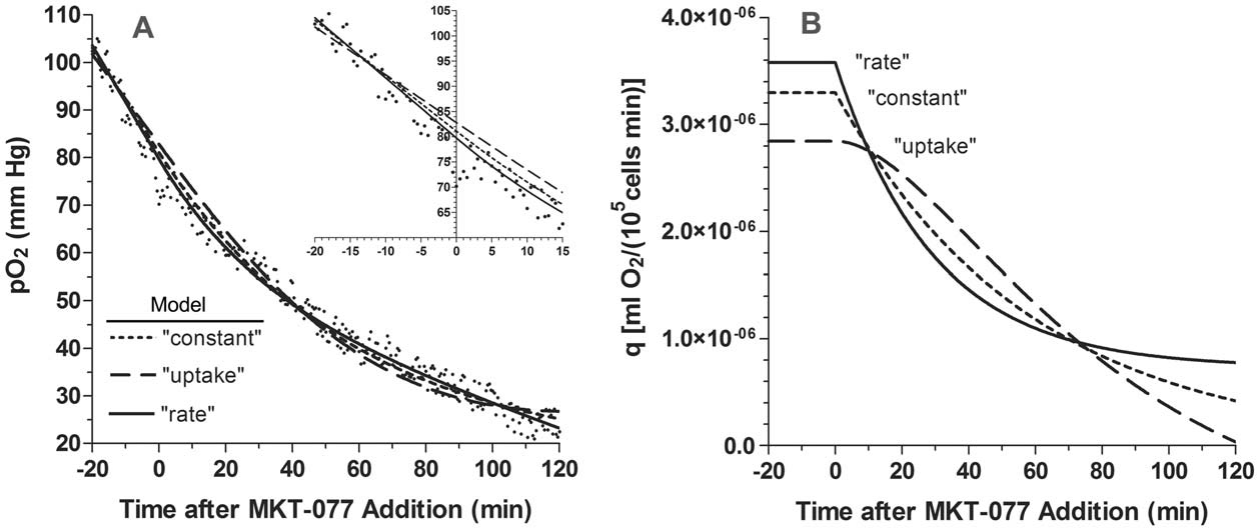
Fits of a typical pO_2_ data set to the three oxygen consumption models. A) Change in pO_2_ measured in R3230Ac cell suspension before and after addition of 6 µg/ml MKT-077 to air-saturated medium at t = 0 minutes. Points are measured pO_2_ data. Curves are fits of data to the following models: dq/dC_C_ = −α_0_ (“constant”, dotted line); dq/dC_C_ = −α_1_C_C_ (“uptake”, dashed line); dq/dC_b_ = −α_2_ (dC_C_/dt) (“rate”, solid line) [see Equations 14–17]. The inset shows the same data from −20 to 15 minutes. B) Corresponding changes in oxygen consumption, q, predicted by the three models (Equations 10–13).

Fits of a representative data set to the three models and the predicted changes in oxygen consumption after addition of 6 µg/ml MKT-077 at time 0 are presented in Figure 3. In this example, the simplest model (“constant”) fitted the data fairly well, but it overestimated the data from 0 to 15 minutes and underestimated it from 60–80 minutes (Figure 3A). The consumption was still decreasing after 120 minutes and seemed to be heading for an unrealistic negative value (Figure 3B). The “uptake” model yielded the worst fit and clearly underestimated the initial consumption rate, q_1_, as evidenced by the shallow slope of the line between −20 and 0 minutes (Figure 3A and inset). The consumption rate had reached zero after 120 minutes and then became negative (Figure 3B). The “rate” model yielded the best fit and described the pO_2_ data reasonably well (Figure 3A). The consumption had almost reached a positive steady state value by 120 minutes (Figure 3B).

The qualitative results evident in Figure 3 were consistent with the quantitative analysis of all 20 fits to the three models. The goodness-of-fit statistics for these fits are summarized in Table 2. A repeated measures one-way ANOVA test of the RMS errors and r^2^ values revealed that there was a significant difference in both parameters among the three models (p<0.0001, n = 20). Subsequent testing using Bonferroni's multiple comparison test revealed that the goodness of fit parameters from the “uptake” model were significantly worse than the parameters obtained from either the “constant” or the “rate” model (p<0.01). The “uptake” model also significantly underestimated the pre-injection consumption rate, which could be calculated from the slope of the points between −20 and 0 minutes (Table 2). Although there was no statistical difference in the goodness of fit parameters between the “constant” model and the “rate” model based on the Bonferroni's test, the simple constant model resulted in unrealistic negative values for q in 12 of the 20 cases (Table 2). In addition, a paired t-test of the RMS errors and the r^2^ values from just the “constant” and “rate” models showed that these parameters were significantly better for the “rate” model (p<0.02). Based on these considerations, the “rate” model was chosen as the best model and was investigated further.

**Table 2:** Comparison of the fits of all 20 data sets to the three different models of oxygen consumption change.

Model	Model Function	r^2^	RMS error (mm Hg)	Fitted parameter q_1_ [ml O_2_/(10^5^ cells min)]	Number of fits with q<0
_“Constant”_	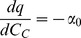	0.9940±0.0034^b^	2.271±0.522^a^	4.14±0.88×10^−6^	5
_“Uptake”_	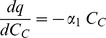	0.9913±0.0062^c^	2.673±0.768^c^	3.79±0.89×10^−6^ d	12
_“Rate”_	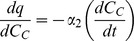	0.9948±0.0024^a^	2.145±0.442^a^	4.32±0.84×10^−6^	0

*Values are means* ± *SD. Repeated Measures One-Way ANOVA: Goodness-of-fit values (r^2^ and RMS error) - p<0.0001. Bonferroni's Multiple Comparison Tests:*

a *p<0.001 vs. “uptake” model value,*

b *p<0.01 vs. “uptake” model value. Paired t-tests:*

c *p<0.02 vs. “constant” model value,*

d *p<0.002 compared to q_1_ values determined from linear fit of pre-injection pO_2_ data (t≤0) {q_1_ = *4.37±1.02×10^−*6*^ *[ml O_2_/(10^5^ cells min)], mean ± SD, n = 20}.*

### Fits of *In Vitro* pO_2_ Data to “Rate” Model

Table 3 summarizes the results from the fits of all of the pO_2_ data to the “rate” oxygen consumption model as defined by Equations 14 and 17. Examples of the fit of this model to pO_2_ data are shown in Figure 2A, and the calculated changes in consumption are shown in Figure 2B. As noted earlier, the control data were fitted to a straight line, and the only parameters obtained were the initial pO_2_, P^*^, and the basal consumption rate, q_1_, which remained constant throughout the experiment. For the MKT-077-treated cells, the r^2^ values show that the model fitted the data well. The r^2^ value for the 2 µg/ml group was slightly higher than the value for the 6 µg/ml group (p<0.05), but all mean r^2^ values were greater than 0.99. There was no difference in the RMS errors, and all values were near 2 mm Hg. The mean 95% confidence intervals were small, demonstrating that the fitted parameters were unique.

**Table 3:** Fitted parameters of the “rate” and “relative rate” models used to fit the pO_2_ data (Equations 14 and 17 or 20).

[MKT-077] (µg/ml)	n	P* Initial pO_2_ (mm Hg)	q_1_ [ml O_2_/(10^5^ cells*min)]	α_2_ {[ml O_2_ (10^5^ cells)]/(ng MKT-077)^2^}	α_r_ {[ml O_2_ (10^5^ cells)^0.5^]/(ng MKT-077)^0.5^}	Calculated q_2_ [ml O_2_/(10^5^ cells*min)]	r^2^	RMS error (mm Hg)
0	6	124.0±19.4*(123.4–124.7)*	3.80±0.75×10^−6^ *(3.75–3.84×10^−6^)*	----	----	----	0.995±0.002	2.15±0.45
2	7	133.7±11.8*(133.2–134.2)*	3.99±0.56×10^−6^ *(3.92–4.05×10^−6^)*	7.53±2.95×10^−7^ *(7.01–8.06×10^−7^)*	5.70±2.23×10^−4^	2.15±0.54×10^−6^	0.997±0.001	1.83±0.24
4	7	132.0±18.2*(131.2–132.8)*	4.90±1.02×10^−6^ *(4.80–5.00×10^−6^)*	3.12±0.89×10^−7^ ** *(2.89–3.36×10^−7^)*	6.68±1.90×10^−4^	1.86±0.61×10^−6^	0.995±0.002	2.27±0.51
6	6	127.6±11.6*(126.7–128.5)*	4.02±0.58×10^−6^ *(3.94–4.10×10^−6^)*	1.34±0.31×10^−7^ ** *(1.28–1.40×10^−7^)*	5.25±1.23×10^−4^	1.09±0.67×10^−6^ *	0.993±0.003*	2.37±0.38
ANOVAp-value	20 or 26	0.682	0.061	<0.0001 (n = 20)	0.385 (n = 20)	0.017 (n = 20)	0.020	0.118
All doses	20 or 26	129.6±15.2	4.20±0.84×10^−6^	4.13±3.18×10^−7^	5.91±1.87×10^−4^	----	----	2.15±0.44

*Control experiments (row 1) were fitted to a straight line. Values are means* ± SD for each parameter. Values in italics are the means of the 95% confidence intervals. P* = initial PO_2_, q_1_ = initial consumption rate, α_2_ = proportionality constant from “rate” model, α_r_ = true proportionality constant ( *Equation 18* *), q_2_ = consumption rate at equilibrium. The p-value reports the significance of the one-way ANOVA test to compare the values among the groups. The Bonferroni t-test was used to compare the multiple pairs among the groups if the ANOVA test showed a significant difference.*

** *p<0.001 vs. 2 µg/ml value;*

* *p<0.05 vs. 2 µg/ml value.*

Among the three fitted parameters, the only significant difference was in the proportionality constant, α_2_ (ANOVA, p = 0.001). The α_2_ value for the 2 µg/ml group was significantly higher than that of either of the other two groups (Bonferroni's test, p<0.001). There was no difference in the starting pO_2_ value, P*, among the groups (ANOVA, p = 0.682). As expected, there was also no difference in the baseline oxygen consumption rate, q_1_, among the groups (ANOVA, p = 0.061), although the mean in the 4 µg/ml dose group tended to be slightly higher.

### Proportionality Constant, α_2_

Since α_2_ was significantly different for the 2, 4, and 6 µg/ml dose groups, the relationship between α_2_ and the concentration of MKT-077 in the media, C_M0_, was investigated further. It was found that α_2_ was approximately proportional to C_M0_^−1.5^. Since, according to Equation 2, C_M0_ is directly proportional to C_C,∞_, α_2_ is also proportional to C_C,∞_^−1.5^ or any combination of C_M0_ and C_C,∞_ to the −1.5 power. Therefore, we defined α_2_ as:
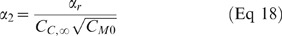
where α_r_ = proportionality constant {[ml O_2_ (10^5^ cells)^0.5^]/(ng MKT-077)^0.5^}. Equation 18 can be used to calculate the new parameter α_r_ for each fit. The average of this parameter for each experimental group is presented in Table 3. There was no significant difference in the new proportionality constant, α_r_, among the experimental groups (ANOVA, p = 0.384). Therefore, unlike α_2_, α_r_ is indeed a true proportionality constant that does not change with drug concentration.

Substituting Equation 18 into Equation 9 yields:
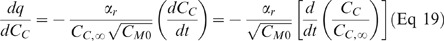
Thus, the relative uptake rate [d(C_C_/C_C,∞_)/dt] is an important parameter in determining the magnitude of the consumption change caused by MKT-077 uptake.

The new constant α_r_ can also be substituted into the expressions describing the pO_2_ change and the consumption change after addition of MKT-077 by inserting Equation 18 into Equations 17 and 13, respectively:
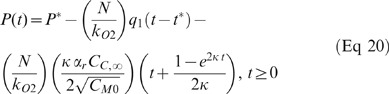


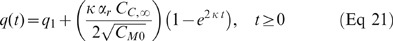



### Effect of Drug Concentration on MKT-077-Induced Metabolic Inhibition

**Figure 4:**
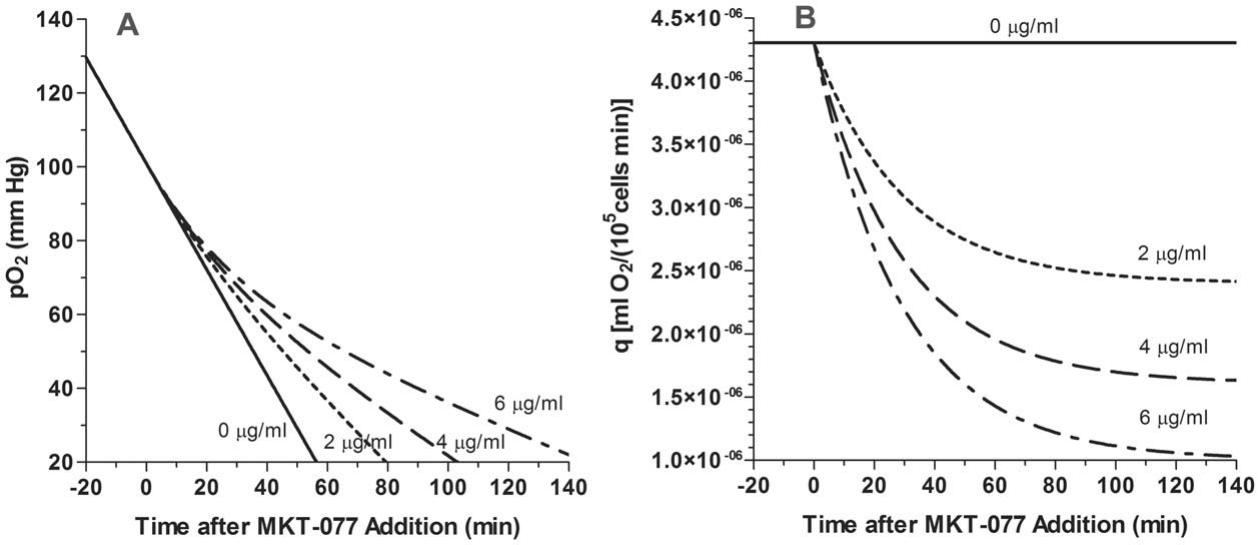
Modeled changes in pO_2_ and predicted oxygen consumption changes after treatment with MKT-077. A) Average pO_2_ measured in R3230Ac cell suspensions before and after addition of different concentrations of MKT-077 (0, 2, 4, or 6 µg/ml) to air-saturated medium at t = 0 minutes. Curves are the “relative rate” model equations (Equations 14 and 20) calculated using the mean fitted parameters (Table 3). B) Predicted oxygen consumption, q, calculated using Equations 10 and 21 and the mean fitted parameters (Table 3).

Equations 20 and 21 can be used to graphically demonstrate the impact of MKT-077 on chamber pO_2_ and cellular oxygen consumption rate by using the mean parameter values in Table 3 (Figure 4). Increasing doses of MKT-077 caused an increased flattening of the pO_2_ curve (Figure 4A), which was the result of a dose-dependent increase in the MKT-077-induced decrease in oxygen consumption rate (Figure 4B).

The quantitative impact of MKT-077 on oxygen consumption is best described by looking at the steady-state values of oxygen consumption. The final steady-state consumption after addition of MKT-077, q_2_, can be calculated from Equation 21 by letting t approach ∞:
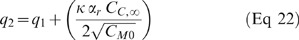



Values of q_2_ were computed from Equation 22 for each experiment, and the group means appear in Table 3. There was a significant difference in q_2_ among the groups (ANOVA, p = 0.017). The q_2_ value was significantly lower in the group treated with 6 µg/ml compared to the 2 µg/ml treatment group (Table 3).

**Figure 5:**
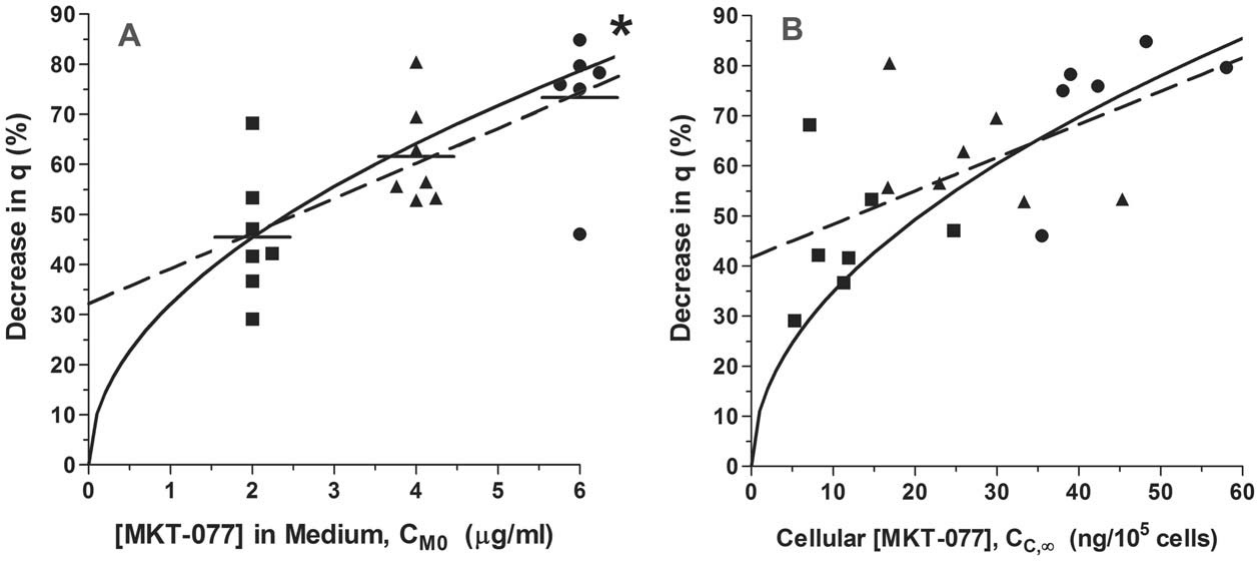
Relative change in oxygen consumption as a function of extracellular and intracellular MKT-077 concentration. A) Percent decrease in oxygen consumption of R3230Ac cells, %Δq, as a function of [MKT-077] in medium. The points are staggered along the abscissa for clarity, and the horizontal bars represent the mean values. The %Δq values were significantly dependent on dose (ANOVA, p = 0.003). The dashed line is a linear regression: %Δq = 6.99C_M0_+32.2, r = 0.706, p = 0.0005, n = 20. The solid curve is the predicted relationship calculated from Equation 24 using the mean parameters. *p<0.01 compared to the 2 µg/ml group using one-way ANOVA with Bonferroni's Multiple Comparison Test. B) Percent decrease in oxygen consumption of R3230Ac cells, %Δq, as a function of steady-state MKT-077 uptake. The dashed line is a linear regression: %Δq = 0.664C_C,∞_+41.7, r = 0.621, p = 0.004, n = 20. The solid curve is the predicted relationship calculated from Equation 25 using the mean parameters. (▪): 2 µg/ml MKT-077, n = 7; (▴): 4 µg/ml MKT-077, n = 7; (•): 6 µg/ml MKT-077, n = 6.

To normalize for variability in the basal consumption rates, the percent decrease in consumption, %Δq, was calculated as:
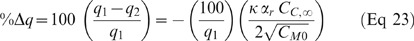



For cells treated with 2, 4, or 6 µg/ml MKT-077, the average decreases in oxygen consumption rate were 45.5±12.6% (n = 7), 61.7±10.2% (n = 7), and 73.3±13.8% (n = 6), respectively (Figure 5A). The percent decrease in consumption was dependent on dose (ANOVA, p = 0.003, Figure 5A), and the percent decrease in consumption in the cells treated with 6 µg/ml was significantly greater than in the cells exposed to 2 µg/ml (p<0.001).

By substituting Equation 2 into Equation 23, %Δq can be expressed as a function of the MKT-077 concentration in the external media, C_M0_:
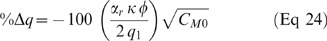
This predicted relationship between the relative consumption change and C_M0_ is shown by the solid curve in Figure 5A.

It is also informative to view the impact of final cellular drug uptake on the total oxygen consumption decrease. For cells treated with 2, 4, or 6 µg/ml MKT-077, the mean intracellular drug concentrations at the end of these experiments were 11.9±6.5, 27.3±10.1, and 43.5±8.4 ng/10^5^ cells, respectively (p<0.0001, ANOVA). There was a significant correlation between the uptake of MKT-077 and percent decrease in consumption (p = 0.004, Figure 5B).

Using Equations 2 and 23, the total decrease in consumption can be expressed in terms of final steady-state concentration, C_C,∞_:
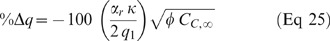
If we assume that the final drug uptake was equal to the final steady-state concentration, C_C,∞_, the relationship in Figure 5B could be predicted by Equation 25. The solid curve in the figure shows the predicted relationship, assuming the same mean parameter values used to fit the data. Given the fact that the MKT-077 concentration at the end of the experiments may underestimate C_C,∞_, the curve describes the data reasonably well.

### Relationship between Transient MKT-077 Uptake and MKT-077-Induced Metabolic Inhibition

**Figure 6:**
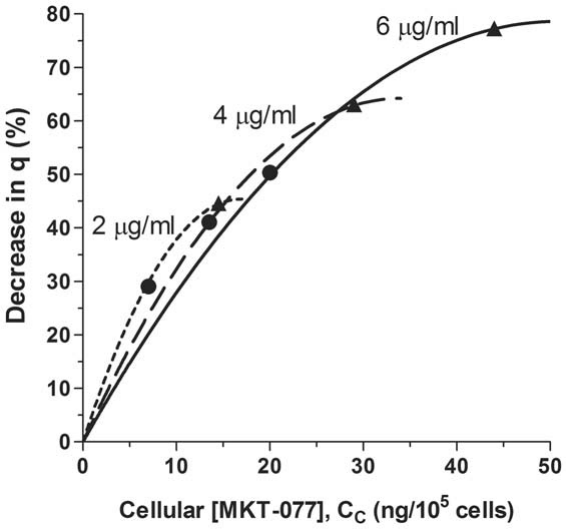
Theoretical relative decrease in oxygen consumption of R3230Ac cells as a function of MKT-077 uptake. R3230Ac cells were treated with 2, 4, or 6 µg/ml MKT-077 at t = 0 minutes. Percent decrease in consumption, %Δq, was calculated from Equation 26 using the mean fitted parameters (Table 3). Points indicate values after 30 minutes (•) or 2 hours (▴) of MKT-077 exposure.

The theoretical relationship between relative consumption change and MKT-077 uptake can be generated by rearranging Equation 3 and substituting the expression for e^κt^ into Equation 21:
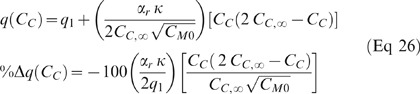



Plots of Equation 26 for the three different doses of MKT-077 are shown in Figure 6 using the mean parameter values generated from the model fits. The steep initial rise in these curves indicates that initial small amounts of drug uptake significantly decrease oxygen consumption. Interestingly, for a given cellular uptake, 2 µg/ml MKT-077 impacts oxygen consumption to a greater extent than 4 or 6 µg/ml, although it takes much longer to achieve a given concentration at the lower doses.

### MKT-077-Induced Metabolic Inhibition of Human Breast Carcinoma Cells

To demonstrate that MKT-077 was also effective at inhibiting oxygen consumption in human breast cancer cells, suspensions of MDA-MB231 cells were exposed to 0 (n = 2), 2 (n = 4), 4 (n = 3), or 6 (n = 2) µg/ml MKT-077 in the metabolic chamber, and the pO_2_ was measured. The intracellular concentration of MKT-077 was determined spectrophotometrically at the end of each experiment, and this final concentration was assumed to be an estimate of the steady-state value, C_C,∞_.

As predicted by Equation 2, the final MKT-077 concentration was strongly positively correlated with the media MKT-077 concentration (r^2^ = 0.908, n = 9, data not shown). The slope of this relationship, φ, was 0.011 ml/(ng MKT-077). Thus, similar to the R3230Ac cells, the MDA-MB231 cells took up MKT-077 in a dose-dependent fashion. Equation 2 was used to calculate the C_C,∞_ values used in subsequent model fits.

**Figure 7:**
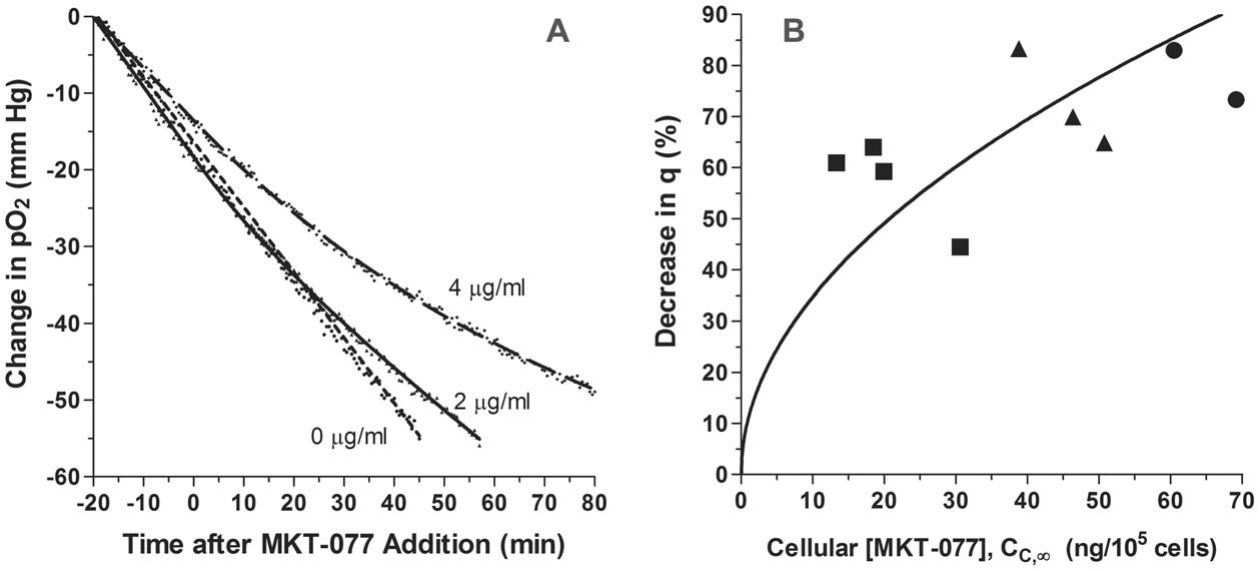
Effect of MKT-077 on pO_2_ and oxygen consumption in MDA-MB-231 cells. A) Change in pO_2_ measured in MDA-MB231 cell suspension before and after addition of different concentrations of MKT-077 (0, 2, or 4 µg/ml) to air-saturated medium at t = 0 minutes. Points are measured pO_2_ data. Curves are fits of data to the “relative rate” model (Equations 14 and 20). B) Percent decrease in oxygen consumption of MDA-MB231 cells, %Δq, as a function of steady-state MKT-077 uptake. The solid curve is the predicted relationship calculated from Equation 25 using the medians of the fitted parameters: q_1_ = 2.91×10^−6^ ml O_2_/(10^5^ cells min), α_r_ = 6.04×10^−4^ ml O_2_ (10^5^ cells)^0.5^]/(ng MKT-077)^0.5^, and κ = −0.101 min^−1^. (▪): 2 µg/ml MKT-077; (▴): 4 µg/ml MKT-077; (•): 6 µg/ml MKT-077.

The pO_2_ profiles were fitted to Equation 20. Since no transient uptake data were available, the parameter κ had to be determined, along with P*, q_1_, and α_r_, using nonlinear least-squares regression (GraphPad Prism). Examples of three pO_2_ curves for the MDA-MB231 cells are shown in Figure 7A. When PBS was added to the chamber at time zero, there was no noticeable change in the slope of the pO_2_ curve. After the addition of 2 µg/ml MKT-077, the slope began to grow shallower, indicating a decrease in consumption. When 4 µg/ml MKT-077 was added, the change in the pO_2_ curve was even more obvious, indicating a larger decrease in consumption. It should be noted that the slope of the 4 µg/ml pO_2_ data is less than the other two slopes before addition of MKT-077, i.e., at times less than zero. The basal consumption rate, q_1_, for these cells was 2.85×10^−6^ ml O_2_/(10^5^ cells min), compared to 3.70 and 4.12×10^−6^ ml O_2_/(10^5^ cells min) for the cells treated with 0 and 2 µg/ml MKT-077, respectively. This fact slightly exaggerates the pO_2_ change for the cells following addition of the 4 µg/ml dose, since the curves do not start at the same ordinate at time zero. Regardless, the functional change and qualitative effect is the same.

Equation 20 described the MKT-077-induced pO_2_ decreases reasonably well (Figure 7A). The mean r^2^ value for all of the fits was 0.996±0.008 (n = 9). There were no differences among the four fitted parameters for the three groups (p>0.12, Kruskal-Wallis test). The mean value of κ, the inverse of the time constant for drug uptake, was −0.013±0.007 min^−1^ (mean ± SD, n = 9). The mean value of the proportionality constant, α_r_, was 5.78±2.23×10^−4^ [ml O_2_ (10^5^ cells)^0.5^]/(ng MKT-077)^0.5^, and the basal oxygen consumption rate, q_1_, was 3.13±1.08×10^−6^ [ml O_2_/(10^5^ cells min)].

As was the case for the R3230Ac cells, MKT-077 caused a decrease in oxygen consumption rate in MDA-MB231 cells (Figure 7B). The mean percent decreases in consumption were 57.2±8.7% (n = 4), 72.7±9.5% (n = 3), and 78.2±6.8% (n = 2) for the 2, 4, and 6 µg/ml doses, respectively. These values were significantly different (Kruskal-Wallis test, p = 0.047), and higher extracellular doses of MKT-077 resulted in more cellular drug uptake, which tended to cause larger decreases in oxygen consumption. The theoretical consumption decrease predicted by Equation 25 is shown in Figure 7B and was calculated using the median parameter values, since the number of experiments was relatively small. The curve matched most of the data reasonably well.

## Discussion

### Modeling of *In Vitro* Cellular MKT-077 Uptake

In this paper, we theoretically modeled the uptake of the lipophilic cation, MKT-077, by R3230Ac rat mammary adenocarcinoma cells and linked the uptake to the driving forces responsible for drug diffusion into the cells. The key parameters are the extracellular MKT-077 concentration, the mass transfer coefficient across the cell membrane (k), the plasma membrane potential, and the mitochondrial membrane potential, which is related to the model parameter β. We tested three extracellular MKT-077 doses, which reflect the lower end of that used during preclinical studies [12], [13], [15], [16]. Not surprisingly, final steady-state MKT-077 uptake was dose-dependent, such that a higher extracellular concentration of MKT-077 resulted in a greater steady-state uptake. Since the time constant describing the drug uptake is the inverse of the parameter κ (Equation 4) and κ was not statistically different among the groups, there was no difference in the time to reach steady state (time constant ∼30 minutes, Figure 1B). Thus, the increased steady-state uptake was entirely caused by the faster uptake rate initially (Figure 1C) and throughout the course of the experiment at the higher drug doses, rather than by an extended uptake time. Since the model parameters associated with cellular transport did not change with MKT-077 dose (Table 1), the difference in uptake rate and final concentration could be entirely attributed to the extracellular concentration differences. Therefore, our modeling showed that the final achievable intracellular concentration was entirely dependent on the extracellular drug concentration and that the drug exposure had no impact on the inherent cellular transport parameters.

Overall uptake of MKT-077 has also been characterized in CX-1 colon carcinoma cells, which took up ∼30 ng/10^5^ cells after two hours of treatment with 3 µg/ml MKT-077 [15]. R3230Ac cells exhibited a similar average uptake after two hours when treated with 4 µg/ml (30.2 ng/10^5^ cells). Based on our modeling, we would expect these two cell lines to exhibit similar transport properties, e.g., similar mitochondrial transmembrane potentials. These intracellular levels of MKT-077 are extremely high and are indicative of the ability of these carcinoma cells to concentrate a lipohilic compound like MKT-077. A concentration of 30 ng/10^5^ cells is approximately 398 µM, compared to the extracellular concentration of 4 µg/ml or 9.3 µM. Thus, the final intracellular concentration is 40–50 times higher than that outside the cell [Equation 2; ratio = (1+β)e^γ^].

Intracellular accumulation of MKT-077 is the result of several different mechanisms. Extracellular MKT-077 enters cells due to concentration and potential differences across the plasma membrane. The MKT-077 that enters the cells either remains free in the cytoplasm or is “bound” to cellular components. This “bound” drug could include drug linked to receptors or drug concentrated within organelles, such as mitochondria. Based on earlier studies that showed very large mitochondrial uptake of MKT-077 [15], [16], we assumed that “bound” drug (C_b_) was primarily attributable to drug sequestered in the mitochondria, i.e., the drug bound to receptors was negligible. The concentration of mitochondrial MKT-077 can be considered proportional to the cytoplasmic concentration (Equation A2) [29]. The model based on these assumptions fitted the data well and showed that the “bound” form accounted for the majority of the large increase in total cellular MKT-077 concentration, while the cytoplasmic concentration (C_i_) contributed little (Figure 1A). The ability of the model to adequately describe the data suggests that the assumptions are reasonable. They are consistent with the idea that the mitochondrial membrane potential is the major driving force for the cellular drug accumulation [15], [16].

Although the mitochondrial membrane potential is most likely the major factor responsible for the large MKT-077 accumulation, MKT-077 certainly binds to receptors within cells and this could also influence the uptake. MKT-077 has been shown to bind to F-actin [30], telomerase [31], and several members of the Hsp70 family, including mortalin (mot-2/mthsp70/GRP75) [32], [33], [34] and HSC70 [30]. Recently, it has been proposed that binding of MKT-077 to these molecules is the basis of its cytotoxic effects, rather than the mitochondrial damage as proposed originally [15], [16], [18]. The exact mechanism responsible for the cytotoxicity is currently unknown, but several hypotheses have been proposed, including general mitochondrial disruption and inhibition of electron transport chain enzymes [15], [16], competitive mot-2 binding that releases p53 [34], suppression of ras transformation by blocking membrane ruffling [30], and inhibition of telomerase activity [31]. The majority of these effects are independent of MKT-077 mitochondrial accumulation and the resultant metabolic inhibition. Thus, as suggested by Naasani et al., the cytotoxic effects of MKT-077 may not be related to the mitochondrial accumulation [31]. While binding of MKT-077 to receptors may play a role in cytoxicity, there is significant evidence that it does not significantly contribute to the large cellular accumulation of the drug. First, confocal microscopy of human pancreatic carcinoma cells exposed to MKT-077 showed that the cells took up large amounts of the drug and that the uptake was almost exclusively localized to the mitochondria [15]. Normal monkey epithelial cells showed no detectable drug in the cytoplasm or mitochondria [15], suggesting that binding to intracellular receptors was below the detection level. Second, simple binding estimates show that this mechanism would contribute little to the overall drug uptake. For example, if we assume that binding between MKT-077 and a receptor is complete and irreversible (an unrealistic, but optimal situation), even a 5 µM receptor concentration would result in a bound MKT-077 concentration of 0.38 ng/10^5^ cells. This would represent only 1.1% of the total MKT-077 in the cells when the extracellular MKT-077 concentration is 4 µg/ml (C_C,∞_ = 33.8 ng/10^5^ cells). Finally, our model described the data well when we assumed that the receptor binding was negligible. Most likely, the receptor binding characteristics would have been nonlinear, rather than the simple constant ratio we assumed for the mitochondrial uptake. Thus, a significant contribution from such nonlinear components would be expected to result in poor model fits. Thus, in agreement with earlier studies [15], [16], [18], our data and model confirm that MKT-077 accumulates to such a large extent in the mitochondria because of the high mitochondrial membrane potential.

Another possible impact of the binding of MKT-077 to receptors could be its possible role in mitochondrial accumulation. Of the currently identified receptors, mot-2 is the only known mitochondrial molecule that interacts with MKT-077. Mot-2 is primarily located in the mitochondria [35], and it can bind to MKT-077 at a single binding site within its p53-binding region [34]. Thus, binding of drug to these molecules could theoretically account for some of the MKT-077 present in the mitochondria. While this could occur, we again would not expect it to significantly contribute to the mitochondrial accumulation, based on the concentration argument presented above. The mot-2 concentration within the mitochondria would have to be 23 µM to contribute 5% of the total MKT-077 concentration. Even if mot-2-binding did significantly contribute to the mitochondrial drug uptake, it would have little effect on the overall interpretation of the results. Its major impact would be that our estimates of mitochondrial membrane potential (ΔΨ_mit_) are too high, since its calculation is based on the assumption that the “bound” drug concentration (C_b_) is completely attributable to drug taken up by the mitochondria as a result of the transmembrane potential (Equation 6). Therefore, it should be noted that the calculated values of ΔΨ_mit_ are estimates, although we believe that they are close to the true values.

In our model, we assumed that the plasma membrane and mitochondrial transmembrane potentials remained constant during the MKT-077 exposure. Since the drug accumulates in the mitochondria, it is possible that the mitochondrial transmembrane potential could change as MKT-077 is bound. However, since the model fitted the data well, we have no reason to doubt the validity of this assumption. In addition, if MKT-077 had an impact on mitochondrial membrane potential, we would have expected to see a significant impact of MKT-077 dose on the fitted values of β and thus mitochondrial transmembrane potential, ΔΨ_mit_. However, there was no difference in β or ΔΨ_mit_ among the dose groups. Finally, three experiments were performed to determine if there was a change in mitochondrial transmembrane potential in the presence of MKT-077. R3230Ac cells were exposed to 6 µg/ml MKT-077 for 0–120 minutes, and mitochondrial potential was assayed using a JC-1 mitochondrial membrane potential assay kit (Cat. No. 10009172, Cayman Chemical Co., Ann Arbor, MI). With this assay the ratio of red-to-green fluorescence is a measure of the mitochondrial membrane potential [36]. MKT-077 had no impact on the red-to-green ratio (data not shown), indicating that the mitochondrial membrane potential was unaffected by MKT-077.

The model described all aspects of the uptake reasonably well, including the time course (Figure 1B) and the magnitude of the final steady-state concentration, C_C,∞_ (Figure 1D). The model tended to underestimate the initial uptake rate of MKT-077 when cells were exposed to 6 µg/ml (Figure 1C). Since the initial uptake rate is proportional to kγ (Equation 5), this product may have been affected by the higher dose of drug. The parameter γ, which related to the plasma membrane potential, ΔΨ_pmem_, was held constant, but the fitted k value tended to be higher in the 6 µg/ml group, but was not significantly different. It is possible that γ or ΔΨ_pmem_ was slightly affected by this high level of MKT-077, which resulted in a small decrease in viability after 90 minutes of exposure. Thus, our assumptions may have resulted in a slight underestimate of the initial uptake rate for the 6 µg/ml dose. Nevertheless, the lack of any statistically significant differences in the parameters shows that the doses of MKT-077 had very little impact on the cellular parameters, including ΔΨ_pmem_ and ΔΨ_mit_.

### Modeling MKT-077-Induced Metabolic Inhibition as a Function of Cellular Drug Uptake

In our experimental system to determine oxygen consumption, it was observed that once the pO_2_ in the chamber dropped below 20 mm Hg, there were changes in cellular oxygen consumption, even in saline-treated controls. This limited the length of time during which reliable data could be obtained for some dose groups. The source of this behavior was most likely the time course of the pO_2_ changes in the system. In one study using rat hepatocytes, when oxygen levels changed quickly (<40 minutes), rates of oxygen consumption did not change until pO_2_ fell below 10 mm Hg [37]. However, when oxygen levels gradually fell over several hours, changes in consumption were sometimes seen at pO_2_ levels as high as 70 mm Hg. Since oxygen levels could adequately be described as falling more gradually in our experiments, decreases in oxygen consumption below 20 mm Hg could be explained by this phenomenon.

Since cell viability remained above 90% throughout all of the experiments, the changes in chamber pO_2_ were the result of MKT-077-induced changes in oxygen consumption rate, rather than a decrease in cell number. There was a small loss of viability when cells were exposed to 6 µg/ml MKT-077 for 90 minutes or more, but this would have had little effect on our results. Since the time constant of the consumption decrease was 60 minutes [1/(2κ)], more than 63% of the consumption change had already occurred before there was any significant cell loss. Thus, the large decreases in oxygen consumption seen in these experiments cannot be explained by loss of cell viability.

Modeling of the pO_2_ data provided detailed information on cellular metabolic changes as a function of drug uptake. Although we originally hypothesized that the consumption change would be proportional to the amount of drug taken up by the mitochondria, this assumption resulted in poor data fits (Figure 3 and Table 2). Surprisingly, the data were best described by a model in which the change in consumption with MKT-077 uptake was proportional to the relative rate of drug uptake (Figures 2 and 3 and Table 2). Based on this model, consumption decreased monoexponentially with a time constant of 1/(2κ) (∼60 minutes, Equation 21). Since the parameter κ is essentially the ratio of the driving force for cytoplasmic MKT-077 uptake to the driving force for mitochondrial drug uptake (Equation 4), the magnitude of the time constant would increase as the mitochondrial transmembrane potential (or β) grows larger. Thus, assuming other parameters remain unchanged, consumption should decrease more quickly in cells with greater mitochondrial transmembrane potentials, i.e., more negative potentials. Therefore, MKT-077 would be most effective in cells with large mitochondrial membrane potentials. This result also suggests that it might be useful to try to increase the absolute mitochondrial membrane potential, i.e., make it more negative, before exposing the cells to MKT-077. If this were possible, such a combination could improve the efficacy of MKT-077 as a potential radiosensitizer.

The change in oxygen consumption was a complex relationship involving a quadratic function of MKT-077 uptake and extracellular drug concentration (Equation 26). As evident in Figure 6, cells that were treated with lower concentrations of MKT-077 required less drug accumulation to achieve the same level of inhibition than cells treated with higher doses. For example, 2 µg/ml MKT-077 reduced oxygen consumption by ∼30% after the accumulation of ∼7 ng/10^5^ cells, while cells treated with 6 µg/ml MKT-077 required an uptake of ∼11 ng/10^5^ cells to achieve the same result. It should be noted, however, that it took 30 minutes of drug exposure at the 2 µg/ml dose to achieve this level of inhibition, while at the higher dose, only about 10–12 minutes of exposure was necessary. Thus, at any given time, higher extracellular drug concentrations resulted in higher MKT-077 uptakes and larger decreases in oxygen consumption (Figures 4B and 6). Nevertheless, this result means that low external MKT-077 concentrations can still be effective at significantly inhibiting consumption, even though a smaller amount of drug accumulates in the tumor. A potential benefit of this feature is that lower external drug concentrations would also be expected to decrease accumulation in normal tissues, and should reduce any normal tissue toxicity. Therefore, in an *in vivo* study, it could be beneficial to infuse MKT-077 at a slower infusion rate, even though it results in a lower plasma drug concentration. This protocol should still decrease tumor oxygen consumption, but may minimize any toxic side effects.

### *In Vitro* MKT-077-Induced Steady-State Metabolic Inhibition

As might be expected, increasing the dose of MKT-077 yielded an increase in final steady-state metabolic inhibition. Somewhat surprisingly, the overall decrease in consumption was not directly proportional to the steady-state intracellular MKT-077 concentration (Figure 5). Instead, our modeling revealed that the change in consumption was actually proportional to the square root of the final, steady-state intracellular MKT-077 concentration (Equation 25). This means that in order to double the decrease in consumption it would be necessary to quadruple the final drug concentration. However, the benefit of this relationship lies in the fact that it increases the impact of small drug accumulations on oxygen consumption. This effect is evident when the slope of the curve in Figure 5B is considered. The changes in consumption for a given change in uptake are greatest at concentrations less than about 20 ng/10^5^ cells. It is also evident that steady-state intracellular MKT-077 concentrations as low as 5 ng/10^5^ cells (50 fg/cell or ∼66 µM) can result in a metabolic inhibition of 20%. Thus, MKT-077 is a powerful inhibitor of oxygen consumption *in vitro*, even at low cellular concentrations.

Our current study is the first to determine MKT-077-induced metabolic inhibition in whole cells. In an earlier study using mitochondria isolated from rat liver and muscle, as well as mitochondria isolated from cultured CV-1 epithelial cells, CX-1 colon carcinoma cells, and CRL1420 pancreatic carcinoma cells, MKT-077 was shown to effectively inhibit mitochondrial respiration in each cell line in a dose-dependent manner [16]. Notably, mitochondria from CV-1 cells required almost four times the MKT-077 dose to exhibit the same level of inhibition as found in CX-1 cells, highlighting the drug's cellular selectivity. While such data are valuable in a mechanistic sense, the use of whole cells offers more meaningful information, since mitochondria can be influenced by other cellular processes and since cellular uptake is governed by transport through the plasma membrane. By using a mathematical model linking the change in oxygen consumption with cellular uptake, we were able to demonstrate the complex, nonlinear relationship between steady-state cellular MKT-077 levels and the extent of metabolic inhibition.

### Comparison of MKT-077 Effects in R3230Ac and MDA-MB231 Cells

Although we did not perform a detailed uptake analysis for the MDA-MB231 cells, data from the consumption experiments yielded information on the nature of MKT-077 uptake by these cells. For example, the parameter φ, the slope of the line relating C_C,∞_ to C_M0_ (Equation 2), was 0.00845 ml/10^5^ cells for the R3230Ac cells, while it was 0.011 ml/10^5^ cells for the MDA-MB231 cells. The higher value of φ for the MDA-MB231 cells indicates that they tended to take up more MKT-077 than the R3230Ac cells at any given extracellular concentration, i.e., C_C,∞_ was larger. Based on Equation 2, this could be caused by a greater mitochondrial transmembrane potential (β) or a greater plasma membrane potential (γ) in the MDA-MB231 cells. From the consumption modeling, we determined a value of κ for the MDA-MB231 cells of −0.013 min^−1^, which was 24% lower than the mean value for the R3230Ac cells (−0.017 min^−1^). Since κ is the inverse of the time constant governing MKT-077 cellular uptake, one might predict that the MDA-MB231 cells would take up the drug more slowly, but the drug uptake rate is dependent on both κ and C_C,∞_ (Equation A14). Since C_C,∞_ is larger for the MDA-MB231 cells, the uptake rate is actually greater for the MDA-MB321 cells compared to the R3230Ac cells at all times after one minute.

MKT-077 resulted in a decrease in oxygen consumption rate in both cell lines. Two-way ANOVA analysis of the percent decrease in consumption data after exposure to 2, 4, and 6 µg/ml MKT-077 for the two cell lines showed a significant impact of MKT-077 dose (p = 0.009). There was no significant effect of cell line (p = 0.065), although there was a tendency for the inhibition in the MDA-MB231 cells to be greater (57 vs 45%, 73 vs 62% and 78 vs 73% at the three doses).

There was no difference between the proportionality constants, α_r_, for the two cell lines (p = 0.870), and both had a value of about 5.8–5.9×10^−4^ [ml O_2_ (10^5^ cells)^0.5^]/(ng MKT-077)^0.5^. Whether this constant would have a similar value for other cell lines remains to be seen. It is not known what factors might impact α_r_.

The MDA-MB231 results show that human carcinomas also take up MKT-077, resulting in inhibition of oxygen consumption. Most likely, MKT-077 would have similar effects on other carcinoma cell lines and could perhaps be effective in other types of tumor cells as well. Clearly, multiple factors impact the uptake, uptake rate, and the magnitude of metabolic inhibition, and these would be expected to vary across cell lines.

### Conclusion - Implications for *In Vivo* Applications of MKT-077

In summary, these findings could have significant implications for *in vivo* applications of MKT-077 or other cationic metabolic inhibitors. First, large amounts of drug do not have to be delivered to achieve a meaningful reduction in oxygen consumption. Second, it might be beneficial to infuse a lower concentration of MKT-077 for an extended period of time, thereby maximizing the consumption change for a given amount of drug uptake and minimizing uptake by normal tissues. Such lower MKT-077 infusion rates might mitigate any deleterious side effects of the drug. In conclusion, MKT-077 shows promise as a metabolic inhibitor and could prove useful as a means to increase tumor pO_2_ levels, thereby increasing the efficacy of radiotherapy.

## Supporting Information

Appendix S1 Supplementary Equations.
(DOC)
