# Supplementary material for: Uptake Rate of Cationic Mitochondrial Inhibitor MKT-077 Determines Cellular Oxygen Consumption Change in Carcinoma Cells
Source: PLoS One. 2012 May 17;7(5):e37471. doi: 10.1371/journal.pone.0037471 (PMC3355126; doi:10.1371/journal.pone.0037471)
Supplement: Appendix S1 — Supplementary Equations. (DOC) [file pone.0037471.s001.doc]

**APPENDIX S1**

*Model of Cellular MKT-077 Uptake*

When MKT-077 is added to cells suspended in medium at time t=0, the drug enters the cells and is either free in the cytoplasm or “bound” to cellular components. This “bound” drug could include drug linked to receptors or drug concentrated within organelles, such as mitochondria. Intracellular drug concentration is described at any time t by:

$C_{C}= C_{i} + C_{b}$ (Eq A1)

where C_C_ = total intracellular MKT-077 concentration, C_i_ = concentration of free unbound MKT-077 in the cytoplasm, and C_b_ = concentration of MKT-077 sequestered within the mitochondria or other organelles or bound to other receptors. All concentrations are expressed in [ng MKT-077/(ml cells)].

Based on previous studies showing large mitochondrial uptake of MKT-077 [15,16], C_b_ is assumed to primarily represent drug concentrated in the mitochondria. Assuming that C_b_ is in thermodynamic equilibrium with C_i_ [29], the binding of MKT-077 is described by:

$C_{b}= \beta C_{i}$ (Eq A2)

where β = equilibrium constant (dimensionless). Therefore:

$C_{C}= (1 +\beta) C_{i}$ (Eq A3)

Since MKT-077 is most likely not a substrate for drug efflux pumps [14], C_C_ can only change when MKT-077 enters the cell by diffusion across the membrane. The change in C_C_ is driven by a combination of the concentration gradient and electrical potential gradient across the plasma membrane. Using the constant field assumption defined by Goldman [25], the transmembrane drug flux can be described by the Nernst-Planck equation:

$$J= \eta\gamma\left(\frac{C_{i}-C_{M} e^{\gamma}}{1 - e^{\gamma}}\right)$$

where J = net flux of MKT-077 into the cells per unit surface area of cell membrane [ng/(sec cm^2^)], η = membrane permeability (cm/sec), and C_M_ = medium MKT-077 concentration [ng MKT-077/(ml medium)]. The parameter γ is given by:

$\gamma= -\frac{zF}{RT} \Delta\Psi_{pmem}$ (Eq A4)

where z = molecular charge number (z = +1 for MKT-077), F = Faraday constant [96.5 J/(mol mV)], R = gas constant [8.314 J/(mol K)], T = absolute temperature (K), and ΔΨ_pmem_ = potential difference between the inside of the cell and the extracellular medium.

The rate of change in C_C_ is given by:

$$\frac{dC_{C}}{dt}= \left(\frac{NA_{c}}{NV_{c}}\right) J= \left(\frac{NA_{c}}{NV_{c}}\right) \left(\eta\gamma\right) \left(\frac{C_{i}-C_{M} e^{\gamma}}{1 - e^{\gamma}}\right)$$

where N = cell concentration [cells/(ml suspension)], A_C_ = surface area of cell (cm^2^/cell), and V_C_ = cellular volume (ml/cell). Assuming that cells are spherical, A_C_/V_C_ = 6/d, where d = cell diameter (cm). Therefore:

$$\frac{dC_{C}}{dt}= \left(\frac{6}{d}\right) \left(\eta\gamma\right) \left(\frac{C_{i}-C_{M} e^{\gamma}}{1 - e^{\gamma}}\right)= k\gamma\left(\frac{C_{i}-C_{M} e^{\gamma}}{1 - e^{\gamma}}\right)$$

where k = mass transfer coefficient across cell membrane = 6η/d (sec^-1^).

Since relatively little drug is taken up by the cells compared to the amount in the medium, C_M_ ≈ C_M0_, where C_M0_ = initial MKT-077 concentration in the medium. Thus:

$\frac{dC_{C}}{dt}= k\gamma\left(\frac{C_{i}-C_{M0} e^{\gamma}}{1 - e^{\gamma}}\right)$ (Eq A5)

Substituting Equation A3 into Equation A5 gives:

$$(1 +\beta) \frac{dC_{i}}{dt}= k\gamma\left(\frac{C_{i}-C_{M0} e^{\gamma}}{1 - e^{\gamma}}\right) = \left(\frac{k\gamma}{1 - e^{\gamma}}\right) \left(C_{i} -C_{M0} e^{\gamma}\right)$$

Integration yields:

$C_{i}= C_{M0} e^{\gamma} \left(1 - e^{\frac{k\gamma t}{(1 +\beta)\left(1 - e^{\gamma}\right)}}\right)$ (Eq A6)

Converting to ng MKT-077/(100,000 cells), Equation A6 becomes:

$C_{i}= 100,000 V_{C} C_{M0}e^{\gamma}\left(1 - e^{\kappa t}\right)$ (Eq A7)

where

$\kappa= \frac{k\gamma}{(1 +\beta)\left( 1 - e^{\gamma}\right)}$ (Eq A8)

Substituting Equation A7 into Equation A3 yields:

$C_{C}= 100,000 (1 +\beta) V_{C} e^{\gamma}C_{M0}\left(1 - e^{\kappa t}\right)$ (Eq A9)

As t approaches infinity, C_C_ reaches a final steady-state value, C_C,∞_ (ng MKT-077/10^5^ cells), and Equation A9 reduces to:

$C_{C,\infty}= 100,000 (1 +\beta) V_{C} e^{\gamma}C_{M0}=\varphi C_{M0}$ (Eq A10)

where

$$\varphi= 100,000 (1 +\beta) V_{C} e^{\gamma}$$

Substituting Equation A10 into Equation A9 yields:

$C_{C}= C_{C,\infty}\left( 1 - e^{\kappa t}\right)$ (Eq A11)

Differentiating Equation A11 with respect to time, t, yields the uptake rate at any time:

$\frac{dC_{C}}{dt}= -\kappa C_{C,\infty}e^{\kappa t}$ (Eq A12)

From Equation A11:

$e^{\kappa t}= \frac{C_{C,\infty}-C_{C}}{C_{C,\infty}}$ (Eq A13)

Substituting Equation A13 into Equation A12 yields:

$\frac{dC_{C}}{dt}= -\kappa C_{C,\infty}\left(\frac{C_{C,\infty}-C_{C}}{C_{C,\infty}}\right)= -\kappa\left(C_{C,\infty}-C_{C}\right)$ (Eq A14)

The initial uptake rate [ng MKT-077/(10^5^ cells min)] is given by:

$\left.\frac{dC_{C}}{dt}\right|_{t=0}= \left.-\kappa C_{C,\infty} e^{\kappa t}\right|_{t=0}= -\kappa C_{C,\infty}= -100,000 \left(\frac{k\gamma}{1-e^{\gamma}}\right) V_{C} e^{\gamma} C_{M0}$ (Eq A15)

*Modeling the Relationship between MKT-077 Uptake and Oxygen Consumption Change*

To quantify the effect of MKT-077 on oxygen consumption, we tested several models relating MKT-077 uptake to the change in oxygen consumption. Since it is reasonable to assume that the change in oxygen consumption rate, q [ml O_2_/(10^5^ cells min)], would be related to the amount of MKT-077 sequestered by the mitochondria or the rate at which MKT-077 was taken up by the mitochondria, we tested the following relationships:

$\frac{dq}{dC_{b}} = -\alpha_{b0}$ (Eq A16)

$\frac{dq}{dC_{b}} = -\alpha_{b1} C_{b}$ (Eq A17)

$\frac{dq}{dC_{b}} = -\alpha_{b2} \left(\frac{dC_{b}}{dt}\right)$ (Eq A18)

where dq/dC_b_ = the change in cellular oxygen consumption for a given change in mitochondrial MKT-077 concentration {ml O_2_/[(ng MKT-077) min]}, α_b0_ = proportionality constant {ml O_2_/[(ng MKT-077) min]}, α_b1_ = proportionality constant {[ml O_2_ (10^5^ cells)]/[(ng MKT-077)^2^ min]}, and α_b2_ = proportionality constant {[ml O_2_ (10^5^ cells)]/[(ng MKT-077)^2^]}. The models represented by Equations A16, A17, and A18 are designated the “constant”, “uptake”, and “rate” models, respectively.

Since total cellular MKT-077 concentration, C_C_, is measured experimentally, these equations can be expressed as a function of C_C_. From equations A2 and A3:

$$C_{b}= \left(\frac{\beta}{1 + \beta}\right) C_{C}$$

$$dC_{b}= \left(\frac{\beta}{1 + \beta}\right) dC_{C}$$

Substituting these expressions into Equations A16-A18 yields:

$\frac{dq}{dC_{C}} = -\alpha_{0}$ (“constant” model; Eq A19)

$\frac{dq}{ dC_{C}} = -\alpha_{1} C_{C}$ (“uptake” model; Eq A20)

$\frac{dq}{ dC_{C}} = -\alpha_{2} \left(\frac{ dC_{C}}{dt}\right)$ (“rate” model; Eq A21)

where α_0_ = proportionality constant {ml O_2_/[(ng MKT-077) min]}, α_1_ = proportionality constant {[ml O_2_ (10^5^ cells)]/[(ng MKT-077)^2^ min]}, and α_2_ = proportionality constant {[ml O_2_ (10^5^ cells)]/[(ng MKT-077)^2^]}, and are defined as:

$$\alpha_{i}= \left(\frac{\beta}{1 + \beta}\right)^{n+1} \alpha_{bi}, i=0, 1, or 2$$

From Equation A12:

$\frac{1}{dC_{C}}= -\frac{1}{\kappa C_{C,\infty}e^{\kappa t}}\frac{1}{dt}$ (Eq A22)

Substituting Equations A11, A12, and A22 into Equations A19-A21 and simplifying yields:

$\frac{dq}{dt} = \alpha_{0}\kappa C_{C,\infty}e^{\kappa t}$ (“constant” model; Eq A23)

$\frac{dq}{ dt} = \alpha_{1} \kappa C_{_{C,\infty}}^{2}e^{\kappa t}\left( 1 - e^{\kappa t}\right)$ (“uptake” model; Eq A24)

$\frac{dq}{ dt} = -\alpha_{2} \kappa^{2} C_{_{C,\infty}}^{2}e^{2\kappa t}$ (“rate” model; Eq A25)

If we assume the recording starts at time t^*^ and MKT-077 is added to the system at time t=0, the oxygen consumption rate can be expressed as a function of time. Before addition of MKT-077, the consumption rate is constant:

$q(t) = q_{1} , t^{*} \leq t\leq0$ (Eq A26)

where q_1_ = basal O_2_ consumption rate [ml O_2_/(10^5^ cells min)]. After addition of MKT-077, the three models given in Equations A23-A25 describe the consumption change. Integration of Equations A23-A25 yields:

$q(t) = q_{1}- \alpha_{0} C_{C,\infty}\left( 1 - e^{\kappa t}\right) , t\geq0$ (“constant” model; Eq A27)

$q(t) = q_{1}- \left(\frac{\alpha_{1} C_{C,\infty}^{2} }{2}\right) \left(1 - e^{\kappa t}\right)^{2} , t\geq0$ (“uptake” model; Eq A28)

$q(t) = q_{1}+\left(\frac{\alpha_{2} \kappa C_{C,\infty}^{2}}{2}\right) \left(1-e^{2 \kappa t}\right) , t\geq0$ (“rate” model; Eq A29)

where q(t) = O_2_ consumption rate as a function of time after addition of MKT-077 [ml O_2_/(10^5^ cells min)].

Equations A26-A29 can be substituted into the oxygen mass balance equation for this closed system:

$k_{O2}\left(\frac{dP}{dt}\right) = -N q(t)$ (Eq A30)

where k_O2_ = oxygen solubility (Henry’s Law constant) of the suspension [ml O_2_/(ml suspension mm Hg)], P = pO_2_ in the media (mm Hg), t = time (min), N = cellular concentration (1x10^6^ cells/ml suspension), and q(t) = oxygen consumption rate defined by Equations A26-A29. By integrating this function, pO_2_ can be expressed as a function of time and three unknown parameters (P^*^, q_1_, and α_0_, α_1_, or α_2_):

$P(t) = P^{*}- \left(\frac{N}{k_{O2}}\right) q_{1}\left( t - t^{*}\right) , t^{*} \leq t<0$ (Eq A31)

$P(t) = P^{*} - \left(\frac{N}{k_{O2}}\right) q_{1}\left( t-t^{*}\right) + \left(\frac{N\alpha_{0} C_{C,\infty}}{k_{O2}}\right) \left[t+\frac{1}{\kappa}\left(1-e^{\kappa t}\right)\right] , t \geq0$ (“constant”; Eq A32)

$P(t) = P^{*}- \left(\frac{N}{k_{O2}}\right) q_{1}\left( t-t^{*}\right) + \left(\frac{N}{k_{O2}}\right) \left(\frac{\alpha_{1} C_{C,\infty}^{2}}{2}\right) \left[t+\frac{\left(1-e^{\kappa t}\right)}{2 \kappa}\left(3-e^{\kappa t}\right)\right] , t \geq0$ (“uptake”; Eq A33) $P(t) = P^{*} - \left(\frac{N}{k_{O2}}\right) q_{1}\left( t-t^{*}\right) - \left(\frac{N}{k_{O2}}\right) \left[\frac{\kappa\alpha_{2}C_{C,\infty}^{2}}{2}\right] \left(t+\frac{1-e^{2\kappa t}}{2 \kappa}\right) , t \geq0$ (“rate”; Eq A34)

where P^*^ = initial pO_2_ at time t^*^ (mm Hg). The equations for t > 0 are only valid for pO_2_ ≥ 20 mm Hg (see text).

The oxygen consumption can also be expressed as a function of intracellular MKT-077 concentration, C_C_, rather than time. Substituting Equation A13 into Equations A26-A28 and simplifying yields:

$q(C_{C}) = q_{1}- \alpha_{0} C_{C} , 0 \leq C_{C} \leq C_{C,\infty}$ (“constant” model; Eq A35)

$q(C_{C}) = q_{1}- \left(\frac{\alpha_{1} }{2}\right) C_{C}^{2} , 0 \leq C_{C} \leq C_{C,\infty}$ (“uptake” model; Eq A36)

$q(C_{C}) = q_{1}-\left(\frac{ \kappa\alpha_{2}}{2}\right) \left[ \left(C_{C,\infty} - C_{C}\right) ^{2}-C_{C,\infty}^{2}\right] , 0 \leq C_{C} \leq C_{C,\infty}$ (“rate” model; Eq A37)

where q(C_C_) = O_2_ consumption rate as a function of total intracellular MKT-077 [ml O_2_/(10^5^ cells min)].
